# MAGPIE: accurate pathogenic prediction for multiple variant types using machine learning approach

**DOI:** 10.1186/s13073-023-01274-4

**Published:** 2024-01-08

**Authors:** Yicheng Liu, Tianyun Zhang, Ningyuan You, Sai Wu, Ning Shen

**Affiliations:** 1 Department of Hepatobiliary and Pancreatic Surgery, First Affiliated > Hospital & Liangzhu Laboratory, Zhejiang University School of Medicine, Hangzhou, 311121 China; 2 College of Computer Science, Zhejiang University, Yuquan Campus, Zhejiang University, Rd Zheda 38, Xihu District, Hangzhou, 310007 China

**Keywords:** Pathogenic prediction, Multimodal annotation, Machine learning, Genomic variation

## Abstract

Identifying pathogenic variants from the vast majority of nucleotide variation remains a challenge. We present a method named Multimodal Annotation Generated Pathogenic Impact Evaluator (MAGPIE) that predicts the pathogenicity of multi-type variants. MAGPIE uses the ClinVar dataset for training and demonstrates superior performance in both the independent test set and multiple orthogonal validation datasets, accurately predicting variant pathogenicity. Notably, MAGPIE performs best in predicting the pathogenicity of rare variants and highly imbalanced datasets. Overall, results underline the robustness of MAGPIE as a valuable tool for predicting pathogenicity in various types of human genome variations. MAGPIE is available at https://github.com/shenlab-genomics/magpie.

**Supplementary Information:**

The online version contains supplementary material available at 10.1186/s13073-023-01274-4.

## Background

The rapid accumulation of the whole genome sequencing (WGS) and the whole exome sequencing (WES) data has led to the discovery of a tremendous number of genetic variations, both pathogenic and non-pathogenic. To aid the assessment and understanding of these variations, population databases such as gnomAD [1], ExAC [2], and ChinaMap [3] have been established. Additionally, genetic disease databases, e.g., ClinVar [4], OMIM [5], and HGMD [6], have also amassed a large amount of information on known pathogenic or benign genetic variations. These databases have been widely used as references in the genetic diagnosis of Mendelian diseases.

Pathogenic mutations that cause Mendelian diseases are known to function through various biological mechanisms and thus have been categorized and studied in different aspects. For instance, exonic mutations based on the protein sequence alterations are categorized into synonymous, missense, stop-gain, stop-loss, frameshift, etc. Synonymous mutations do not alter protein sequence, whereas missense mutations result in different amino acids being encoded. Since missense mutation-associated changes in protein sequence may potentially be pathogenic, various studies have focused on predicting the pathogenic impact of missense variants [7–17]. On the other hand, some mutations are pathogenic at the RNA level through splicing alterations, and these mutations are often located in the splicing donor, acceptor, and intronic regions. Consequently, splicing alterations have also been considered for the pathogenicity evaluation of mutations [18, 19]. However, in clinical practice of genetic diagnosis using WES, different types of mutations and mechanisms should be considered simultaneously to identify the pathogenic mutation.

With the development of machine learning (ML) and deep learning (DL), many computational methods using ML or DL have been developed for predicting mutation disruption or pathogenicity. Some methods were developed based on specific biological mechanisms or data types. For example, SpliceAI employs a deep neural network to learn information about splicing codes of the genome and predict whether a mutation affects splicing [19]. Frazer et al. proposed the evolutionary model of variant effect (EVE) based on a deep generation model of evolutionary data to predict the pathogenicity of human missense variants [16]. On the other hand, some algorithms consider ensemble features from multiple aspects and build on top of existing pathogenicity prediction. For example, the Combined Annotation-Dependent Depletion (CADD) implements a support vector machine with annotation features in conservation metrics, regulatory information, transcript information, and so on [20]. Daniel et al. proposed DANN, which uses the same features as CADD but integrates them into a deep neural network for better performance [21]. REVEL is another approach proposed by Nilah et al. that uses a logistic regression model and relies on multiple pathogenicity prediction tools, including MutPred [22], VEST [14], PROVEAN [9], Mutation Assessor [11], and phastCons [23]. Although the aforementioned methods are widely used or developed with state-of-the-art methods, they only apply to specific cases or depend on multiple prediction tools, leaving many genetic variants unpredictable in real-world prediction tasks.

In this study, we present MAGPIE (Multimodal Annotation Generated Pathogenic Impact Evaluator), a pathogenicity prediction tool for all nonsynonymous exonic variants. MAGPIE employs multimodal annotation to annotate all exonic variants to cope with various mutation types and pathogenic mechanisms. The idea is that from the user’s perspective, the pathogenicity of a variant should be jointly evaluated on multiple scopes, and MAGPIE can help automate this process by leveraging the modern machine learning methodologies. We benchmarked MAGPIE against 14 previously published methods and found that MAGPIE outperformed all other methods in both independent test set and several imbalanced orthogonal validation sets. Notably, MAGPIE was able to make predictions on multiple types of exonic mutations, fulfilling 5–60% of unapplicable missing values based on previous methods. Most importantly, the superior performance of MAGPIE in highly imbalanced validation dataset, as well as variants with low population allele frequency highlights its advantage in clinically relevant applications of interpreting VUS for individual patients, where the model is typically applied to identify less than 5% pathogenic variants from tens of thousands of candidate variants. We envision that MAGPIE will be a valuable tool and widely adopted for pathogenicity prediction in the field. MAGPIE is available as an online server at http://tools.shenlab-genomics.org/tools/MAGPIE.

## Methods

### Data preparation

We ensemble several germline mutation databases as the source of our datasets. Germline mutations are changes to DNA that individuals inherit from the egg and sperm cells during conception. ClinVar database (https://ftp.ncbi.nlm.nih.gov/pub/clinvar/, accessed 2022.6.24) was downloaded and included nonsynonymous SNV (missense variants), stop-gain variants, start-loss variants, frameshift mutations, nonframeshift mutations, and stop-loss variants [4]. Variants were categorized as benign, including Likely_benign, Benign, and Benign/Likely_benign labels, and pathogenic, including Likely_pathogenic, Pathogenic, and Pathogenic/Likely_pathogenic, which were selected as true negative (benign) and true positive (pathogenic) labels respectively. All variants with conflicting interpretations and unknown labels were removed. After filtering, we initially identified 78,089 mutations from ClinVar.

To improve the model performance in classifying rare variants, we considered adding rare benign variants to the training dataset as a trade-off between preserving the importance of allele frequency information and helping identify rare pathogenic variants from rare benign variants. We selected ultra-rare benign variants with allele frequency between $1{e}^{-5}$ and $1{e}^{-3}$ based on the gnomAD database (https://gnomad.broadinstitute.org/downloads/, accessed 2022.09.14) [1]. For most chromosomes, there existed about 500,000 available variants. Secondly, we randomly choose 5000 per chromosome. Thirdly, we filtered out variants without enough information after annotating by ANNOVAR (ANNOtate VARition). Finally, we randomly chose again and just retained 500 variants per chromosome. For chr11 and chrY, which did not contain enough variants, we kept them all and randomly used qualified ones in other chromosomes to fill the gap. In total, we incorporated 11,998 variants from gnomAD, and these variants were split into two subsets based on the proportion of the number of variants in ClinVar to that in SwissProt [23] for further training and evaluation. We aim to enhance the model’s ability to deal with rare benign variants; thus, it is crucial to minimize the false positive rate influenced by the allele frequency to identify pathogenic variants in the clinical scenario.

We constructed an orthogonal validation set to evaluate the performance of our model. The orthogonal test set was constructed from SwissProt (https://ftp.uniprot.org/pub/databases/uniprot/, accessed 2022.7.10) [23], which contains over 70,000 variants with validated pathogenicity labels. We selected exonic mutations, filtered out variants labeled as US (uncertain significance), and applied the filtering and transformation process to remove all synonymous SNVs, following the same steps used in the ClinVar training and test datasets.

We obtained the ACMG-guided dataset (10.1016/j.gim.2021.11.018, accessed 2023.05.22), which includes a total of 1270 mutations, from the official website as another orthogonal test panel. We first removed 328 mutations without clear clinical significance labeled as uncertain significance—insufficient evidence or uncertain significance—conflicting evidence, 93 variants whose position cannot be mapped from HGVS ID, 358 intronic, 3′ UTR, and 5′ UTR variants, splice sites, and synonymous SNVs, etc., to ensure a fair comparison with other methods (Additional file 1: Table S3). After filtering, a final dataset of 491 missense mutations was used to compare MAGPIE and other model performances.

Gene-level features encapsulate characteristics that are relevant to genes and are shared across variants within the same gene. As we used gene-level features, variants in the same gene share identical scores for these gene-level features, and it would cause some potential biases and label leakage. Therefore, we performed a random split according to genes. Let $D$ as the dataset to be split; $A$ and $B$ are datasets after splitting; ${L}_{A}$, ${L}_{B}$, and ${L}_{D}$ are lists containing genes for datasets $A$ and $B$; and let $D$ and $N$ as the proportion threshold of variants that should be added to $A$ and ${n}_{A}$ as the realtime proportion of $A$ accounts for $D$. After completing these steps, the resulting datasets would be as follows: ${L}_{A}$ contains the randomly selected genes from ${L}_{D}$. ${L}_{B}$ is the remaining genes in ${L}_{D}$ after removing the selected genes. Datasets $A$ and $B$ consist of mutations occurring in genes belonging to categories ${L}_{A}$ and ${L}_{B}$ within dataset $D$, respectively. We defined the splitting process in Algorithm 1:

**Figure:**
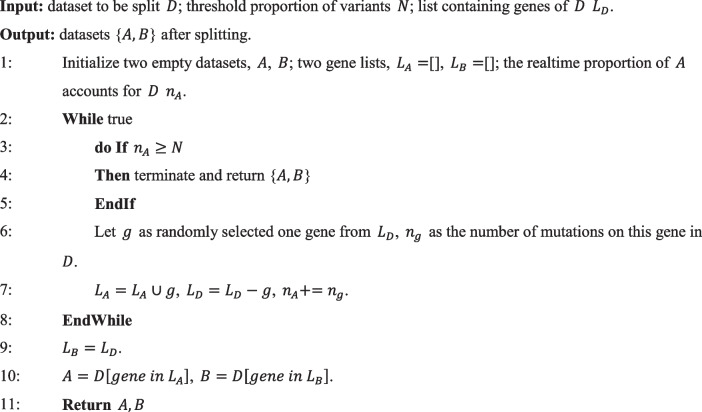
**Algorithm 1.** Dataset split

First, to further enhance the robustness of MAGPIE and ensure a fair comparison, we split the gnomAD dataset. Let ${n}_{ClinVar}$, ${n}_{SwissProt}$, and ${n}_{gnomAD}$ denote the number of variants in ClinVar, SwissProt, and gnomAD, respectively. We define dataset $D$ as gnomAD, threshold $N$ as ${n}_{ClinVar}/\left({n}_{ClinVar}+{n}_{SwissProt}\right)*{n}_{gnomAD}$, and randomly split $D$ into $A$ and $B$ which are then added to ClinVar and SwissProt, respectively. We named these two datasets as ClinVar_gnomAD and SwissProt_gnomAD. Second, we defined dataset $D$ as $ClinVar_{gnomAD}$, threshold $N$ as $\left({n}_{ClinVar}+{n}_{ClinVar}/\left({n}_{ClinVar}+{n}_{SwissProt}\right)*{n}_{gnomAD}\right)*10\%$ and randomly split $D$ into $A$ and $B$, namely training and independent test set respectively. Finally, we excluded all variants in $SwissProt_{gnomAD}$ located on genes that appear in ClinVarTraining and defined it as an orthogonal validation set.

### MAGPIE framework

The machine learning component of MAGPIE is based on a gradient-boosting tree-based model of classifying pathogenic and benign variants, which includes three steps. First, we annotated candidate SNVs to obtain information needed for model training. Second, we used automated feature engineering to pull out meaningful features from the datasets, and then we designed a feature selection strategy to obtain the optimal combination of features to feed the model. Finally, we trained the model using the processed dataset with step-wise tuning to make the process controllable and avoid overfitting.

The whole framework in this paper was mainly implemented in Python (v3.7) [24] and MATLAB [25], and other models were built using the sklearn (v0.21.3) package [26].

### Feature annotation

We annotated candidate SNVs with ANNOVAR (24 October 2019, latest version) [27] and included predicted scores provided by SpliceAI [19].

As for feature selection, we used ANNOVAR, SpliceAI, and the ChromHMM [28] model to annotate candidate variants and ended up with 132 features. To narrow down the number of features, we applied several models on the ClinVar dataset with all these features and kept the models that satisfied over 95% accuracy on the validation set. Then, we calculated feature importance of all input features based on these models and filtered out features whose contribution to the models is less than $1{e}^{-3}$. The number of features was reduced to 60 and below. We will describe these features in detail (Additional file 1: Table S4-S5).

For further training, we included six different types of variant classifications, namely nonsynonymous SNV (missense), startloss, stopgain, stoploss, frameshift, and non-frameshift. For each type of variant, 60 features were retrieved from the annotated datasets, including six categories: (1) epigenomics, (2) functional effects, (3) splicing effects, (4) population-based features, (5) biochemical properties, and (6) conservation (Additional file 1: Table S4-S5). And in our model, we excluded all predicted scores from variant pathogenicity prediction tools to minimize the effects of prior bias.

Epigenomics of each variant was annotated by the 15-state ChromHMM model across nine different cell lines in order to capture the significant combinatorial interactions between different chromatin marks in their spatial context (chromatin states). Functional effects included the gene damage index (GDI), residual variation intolerance score (RVIS), gene intolerance scores based on the loss of function tool (LoFtool), variant types, and annotations from the OMIM database. We use variant types as features including missense, frameshift, nonframeshift, startloss, stopgain, and stoploss. We use the OMIM database to annotate the mutation inheritance pattern, which is categorized into five distinct types: autosomal recessive, autosomal dominant, X-linked recessive, X-linked dominant, and others. Epigenomics features, variant type features, and mutation inheritance pattern features are one-hot encoded and fed into the model for training.

SpliceAI annotated each variant with its predicted effect on splicing. The delta score of the variant represents the probability of the variant being splice-altering as acceptor gain (DS_AG), acceptor loss (DS_AL), donor gain (DS_DG), and donor loss (DS_DL). Furthermore, we also obtained information about the location where splicing changes relative to the variant position, which included the delta position of acceptor gain (DP_AG), acceptor loss (DP_AL), donor gain (DP_DG), and donor loss (DP_DL). SpliceAI does not annotate variants if they are close to chromosome ends or too long reference sequences. We use 0 to fill in missing values of SpliceAI prediction.

Population-based features represented the incidence of an allele in a population, including 12 different types of allele frequencies (AF). We retrieved AF in various populations: all exome, raw allele frequency (AF_raw), African (AF_afr), Latino/Admixed American (AF_amr), Ashkenazi Jewish (AF_asj), East Asian (AF_eas), Finnish in Finland (AF_fin), Non-Finnish European (AF_nfe), and other (AF_oth). Besides, allele frequencies in different genders were also obtained from annotated information.

Biochemical properties contain the effects of amino acid changes before and after mutations. We first checked whether the mutation causes an amino acid change, and if not, we assigned 0 to all relevant features. Amino acid change (AAchange) was used as the basis for subsequent annotation. We stored the physicochemical properties of each amino acid, including molecular weight, equipotential point, dissociation constant, hydrophilicity, polarity, acid–base, etc., in a matrix, in which the characteristics of Boolean type were represented by 1/0. We obtained the corresponding properties of the amino acids by querying the matrix and took the delta value between the properties before and after the mutation as the features of the mutation. We then employed different strategies for different types of variants since some variants cause multiple amino acid changes, which require specific handling. When a mutation affects more than one amino acid, we calculate the average value before and after the change, respectively, to ensure that the feature selection process is comprehensively representative. We also used the information obtained from the BLOSUM100 matrix as a feature to demonstrate the conservation and similarity of amino acid substitutions.

Conservation scores included phastCons 20way mammalian, 30way mammalian, 100way mammalian, 100way vertebrate; phyloP 20way mammalian, 30way mammalian, 100way vertebrate; and odds ratios of SiPhy 29way mammalian.

We use the Bayesian PCA-based missing value estimation method by Oba et al. [29] to impute missing values for several features in preparing the next steps, and any outliers that may arise from extensive imputation are carefully removed from our analysis.

### Feature engineering and selection

We applied automated feature engineering on our training dataset to generate tens of thousands of candidate features to capture potential non-linear and more complex relationships within the data.

We first generate new features based on mathematical transformations of the existing numerical features. AutoFE (automatically feature engineering) applied operations such as logarithms, square roots, and exponentials to the original features. Next, we created new categorical features by grouping the original features based on their values. For example, we grouped conservation scores into categories by binning. These categorical features could capture relationships that might not be easily captured by the original numerical ones. We use the openFE [30] package to perform autoFE.

After generating these new features, we used the default method defined in openFE to perform feature selection to remove any redundant or irrelevant features. OpenFE evaluated the importance of each feature and removed those that did not contribute significantly to the model’s predictive power. Even though the dataset still contains over 3000 features for each variant, which could lead to over-fitting. So, we stepped further based on openFE to perform separated feature selection (SFS).

In our study, we segregated all features into two categories, namely a core set and an add-on set. The former included features related to pathogenicity with validated evidence, such as population-based features, conservation, and functional effects. These features were retained during the training phase. The latter comprised additional features, including biochemical properties, splicing effects, epigenomics, and automated feature-engineered features. We then employed a feature selection process to determine the most important ones for feeding our model. We initially trained a simplified tree-based model for less than 50 rounds to reduce the features to 200 or less. Subsequently, after each round, we evaluated the importance of each feature and discarded those with relative importance scores lower than $1{e}^{-3}$.

After analysis, we observed that the core features exhibited prominent significance compared to the entire set of features. To further enhance the performance of our model, we conducted training using feature combinations, as described in Algorithm 2, to attain the optimal amalgamation of features that would yield superior performance on the test set.

**Figure:**
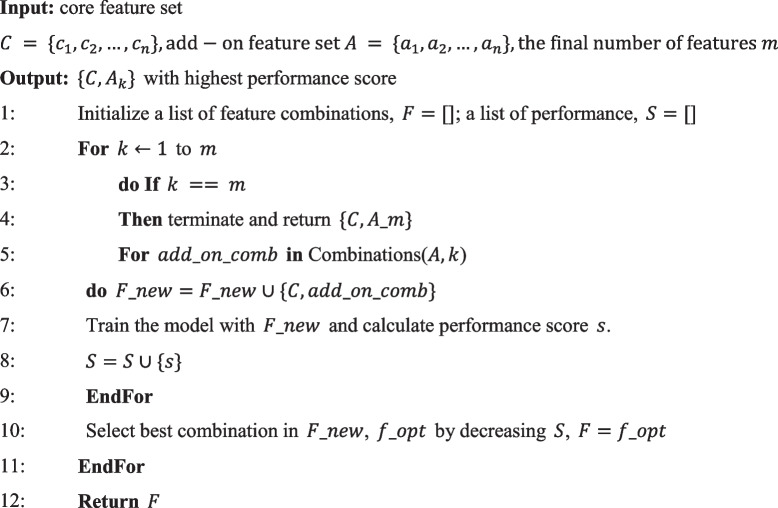
**Algorithm 2.** Separated feature selection

### Model training

MAGPIE was based on a LightGBM (LGBM) model for predicting pathogenicity. LightGBM has a faster training speed and lower memory consumption than other tree-based models. To better learn from data and avoid over-fitting problems, especially as a tree-based model, we defined five rounds to train the model with 5-fold cross-validation in each round.

We first set the initialization parameters of the model, where boosting type is set to gbdt, and the learning rate was set to 0.1. The purpose of the first round was to improve the accuracy, so in this round, we mainly searched for the depth of trees (3–8) and the maximum number of leaf nodes per tree (5–100). Meanwhile, we set early-stopping to 50 to minimize the magnitude of overfitting. The next four rounds of tuning were to make a trade-off between reducing the overfitting and ensuring accuracy after the first round of tuning. In the second round, we adjusted *max_bin*, the number of segments in the histogram algorithm, to discretize the eigenvalues. The value of *min_data_in_leaf* depended on the number of sample trees and *num_leaves* in the training data. Setting it larger can avoid generating a too-deep tree. The third round of adjusting the focus on *feature_fraction* and *bagging_fraction* was to specify a certain percentage of samples from all data for training, which can reduce the variance at the cost of increasing the bias. So, in this step, we tried to reduce the sampling proportion as much as possible while ensuring accuracy. In the fourth round of adjustment, *lambda_l1* and *lambda_l2* were tuned, representing the L1 and L2 regularization terms, respectively, which were used to filter the features and control their influence in the model to prevent some features from greatly affecting the whole model. Finally, we adjusted *min_split_gain*, which means that node splitting will only be performed when the gain is more significant than our given threshold, which will greatly limit the growth of trees. Upon completing the training process, we observed no substantial decrease in accuracy and classification performance measures. This outcome indicates the generalization ability of the model extends far beyond the boundaries of the training dataset, thereby mitigating the issue of learning bias commonly associated within datasets.

### Evaluate and compare models by multiple metrics

To quantitatively evaluate model performance, we compared MAGPIE with 14 other predicted tools including ClinPred [31], REVEL [17], MetaSVM [13], MetaLR [13], VEST4 [14], M-CAP [32], MutationAssessor [11], PrimateAI [33], SIFT4G [34], LIST-S2 [35], DANN [21], MutationTaster [36], VARITY [16], and MutPred [22]. SIFT4G score less than 0.05 is putatively pathogenic according to the authors’ recommendation. For other tools that predict continuous values or probabilities of pathogenicity, we utilized the threshold recommended by the authors in the corresponding article to distinguish between the pathogenic and benign variants. 0.025 is used for M-CAP, 0.8 for Mutation Assessor and PrimateAI, 0.85 for LIST-S2, 0.3 for MutationTaster, 0.79 for MutPred, and 0.5 as the threshold for other prediction tools. Variants with scores greater than or equal to the threshold in the prediction results of each tool possessed to be pathogenic, and those less than the threshold were benign.

Due to the different distribution of pathogenic and benign mutations in both the balanced test set and the imbalanced orthogonal set, we used several different metrics to assess the predictive performance of the model, including accuracy, precision, recall, specificity, F1-score, G-mean, Matthew’s correlation coefficient (MCC). Compared with accuracy and F1-score, MCC, which considers all components in the confusion matrices, can be used even if datasets are very imbalanced. We illustrated curves and computed the area under the receiver operating characteristic (ROC) curve (AUC).

$${\text{accuracy}}=\frac{TP+TN}{TP+FP+TN+FN}$$

$${\text{precision}}=\frac{TP}{TP+FP}$$

$${\text{recall}}=\frac{TP}{TP+FN}$$

$${\text{specificity}}=\frac{TN}{TN+FP}$$

$${\text{Fbeta}}-{\text{score}}=\frac{(1+{{\text{beta}}}^{2})({\text{recall}}\times {\text{precision}})}{{\text{recall}}+{\text{precision}}\times {{\text{beta}}}^{2}}$$

$$g-{\text{mean}}=\sqrt{{\text{recall}}\times {\text{specificity}}}$$

$$mcc=\frac{TP\times TN-FP\times FN}{\sqrt{(TP+FP)(TP+FN)(TN+FP)(TN+FN)}}$$

$${\text{AUC}}=\frac{{\sum }_{i=1}^{n}{\text{Ran}}{k}_{i}-\frac{n\left(n+1\right)}{2}}{{n}_{{\text{positive}}}\times {n}_{{\text{negative}}}}$$

Here, $n$ is the total number of samples, ${n}_{positive}$ is the number of positive samples, ${n}_{negative}$ is the number of negative samples, and $Ran{k}_{i}$ is the rank of the $i$-th sample’s predicted value among all samples. For samples with the same predicted value, their ranks are averaged.

Most models are designed to handle specific variant types or rely heavily on crucial information such as protein structure predictions. As a result, if a variant falls outside the model’s scope or crucial information is unavailable, the pathogenicity prediction becomes challenging. To assess the performance of these prediction tools, we excluded variants without available results for each tool and generated a confusion matrix using the true labels to derive relevant metrics. In addition, we also compared the performance of these tools on unpredictable variants by classifying the variants without predicted results as benign.

### Feature importance

The utilization of autoFE has facilitated the incorporation of numerous novel features, which are the culmination of distinct amalgamations of two distinct features or the distribution of one specific feature. In the case of the original features, the importance is derived from LightGBM. As for the combined features, which were hard to interpret in biology, we employ an average importance metric based on the number of contributing features, which is subsequently added to the original features. Ultimately, we determine the importance of each original feature, followed by the comparison and analysis.

### Website

The accompanying web services of MAGPIE have been developed as shown in Additional file 2: Fig. S1. The tool provides online search functionality for pathogenicity scores by entering information on possible single-nucleotide variants (SNVs). Additionally, users can register and request analysis for non-SNV mutations. The development of MAGPIE was initiated to enhance the precision and convenience of mutation pathogenicity prediction, providing comprehensive support to researchers and clinicians within the biomedical field.

Users of the MAGPIE tool can input mutation information in the chr:start–end-ref-alt format via the website to obtain pathogenicity scores. For bulk variant prediction, users can submit tasks by uploading a csv file. A task ID will be generated upon successful submission, enabling users to obtain pathogenicity annotations from the download page once the prediction process is complete. The tool aims to contribute to advancing research and treatment in this area.

## Results

### MAGPIE overview

**Fig. 1:**
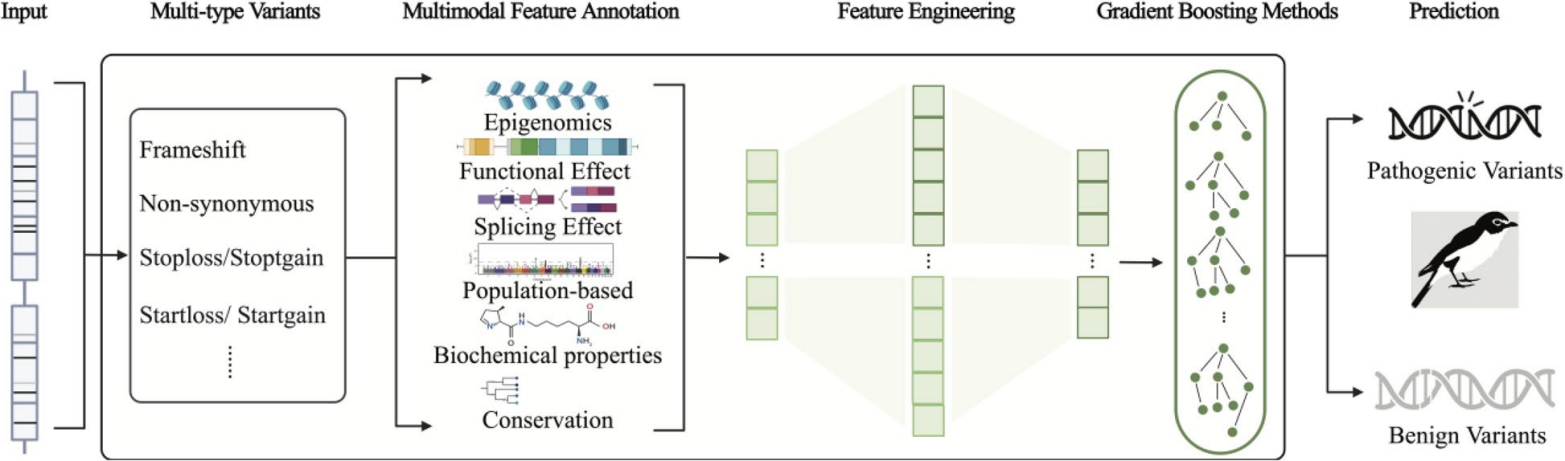
Framework of MAGPIE. The model was trained to predict pathogenic scores of multi-type variants and included three steps. First, candidate variants were annotated with high-dimensional features covering six different modalities. Second, automatic feature engineering and separated feature selection were undertaken step by step. Finally, a gradient boosting method with controllable tuning was implemented to train the model and obtain predictions for the pathogenicity of variants

MAGPIE is a computational method to predict the pathogenicity of multiple variant types using a gradient-boosting machine learning framework (Fig. 1). To prepare data used for modeling, we collected all mutations with a pathogenic interpretation in the ClinVar database and selected mutations labeled as pathogenic (including likely pathogenic) and benign (including likely benign). We then randomly selected 90% of the dataset to use as training data, rendering a total of 39,893 pathogenic and 38,125 benign variants to feed into the model.

The MAGPIE framework is composed of 3 steps (Fig. 1). First, for a given variant, MAGPIE annotates the variant with high dimensional features covering six different modalities, including epigenomics, functional effects, splicing effects, population-based features, biochemical properties, and conservation. In the second step, we use automatic feature engineering followed by feature selection to create candidate features based on the training dataset. Automatic feature engineering aims to create multiple new combinations of features based on input features automatically to capture as much information as possible. After that, feature selection is used to pull out meaningful features. Lastly, using 5-fold cross-validation and step-wise parameter tuning, we trained an interpretable gradient-boosting model (GBM) based on ensemble trees [37] and predicted the probability of pathogenicity in candidate variants. The gradient-boosting model, namely LightGBM, uses algorithms like gradient-based one-side sampling (GOSS) and exclusive feature bundling (EFB) to accelerate the training process while assuring accuracy. The output of MAGPIE is a score of pathogenicity defined over the interval between 0 and 1, in which 0 refers to benign variants and 1 represents the most pathogenic. To understand how MAGPIE achieves satisfying performance with multiple modalities, we performed an ablation study to investigate the effects of the training dataset, feature selection, and LightGBM on MAGPIE. Moreover, the ablation study indicated that the MAGPIE integrating a series of modules achieved the best performance improvement in pathogenic prediction (Additional file 2: Fig. S2). A more detailed description of the MAGPIE framework is included in the “Methods” section. Additionally, we made MAGPIE available through an online website to facilitate access and use of the tool at http://tools.shenlab-genomics.org/tools/MAGPIE (Additional file 2: Fig. S1).

### MAGPIE is interpretable

**Fig. 2:**
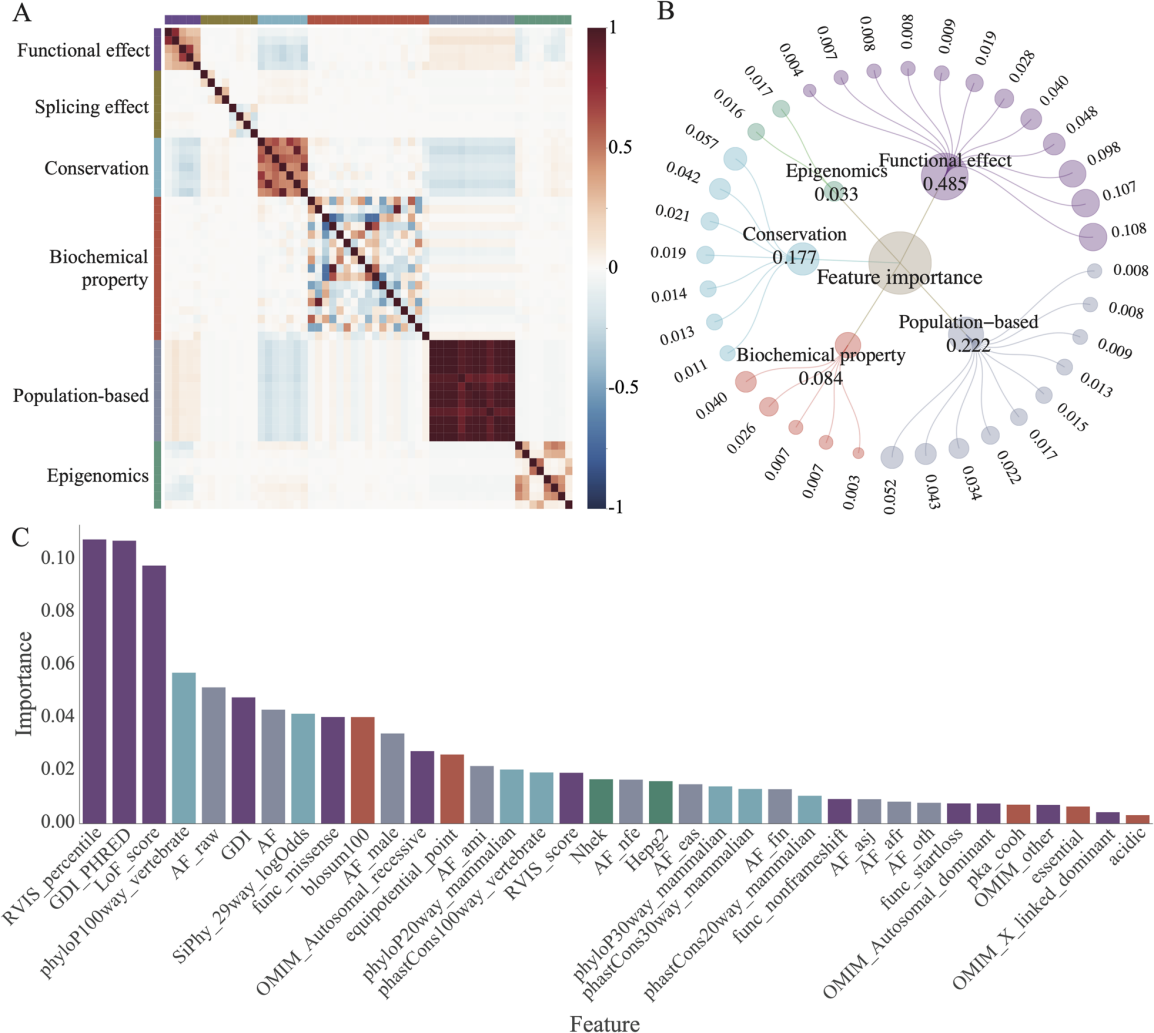
Feature importance and correlation. **A** Correlation between features used to train MAGPIE. **B** A captivating hierarchical relationship diagram is presented, displaying the intricate relationship between features and the categories they belong to. Each dot in the outermost layer represents a distinct feature, while the size of the dots indicates their importance. The second layer depicts feature categories, with the size reflecting the sum of importance of the subordinate features. **C** The bar plot illustrates feature importance, which shows the contribution of each feature after feature selection. Part of the add-on features is automatically removed during the training process

The application of the gradient boosting approach makes MAGPIE an interpretable machine-learning model. To better understand the learned representation, we calculated the importance of different feature modalities and presented them in a network view (Fig. 2B). The feature importance in MAGPIE indicates the degree to which each feature contributes to the model and reflects their information gains. During the classification process, MAGPIE ensembled six modalities of features. Among them, the most important feature group is the functional effect, which includes measures such as the loss of function score (LoF_score), human gene damage index (GDI), and so on (Fig. 2A, B, Additional file 1: Table S4-S5). Loss-of-function mutations have a greater likelihood of causing disease [38]. As previous studies have shown, we found that pathogenic variants predicted by MAGPIE have significantly lower LoF scores than benign variants (Additional file 2: Fig. S3A). GDI is the accumulated mutational damage of each protein-coding human gene, and variants in highly damaged genes are less likely to be disease-causing. We observed a high weight assigned to the GDI feature, indicating that genes with a lower GDI tended to have higher MAGPIE prediction scores and were more likely to be pathogenic (Additional file 2: Fig. S3B), consistent with previously published studies [26]. The second most important feature modality is population-based, which is correlated inversely to the pathogenicity in test datasets. The third most significant feature modality is conservation, which has been considered in many previous methods such as CADD, SIFT [7], PolyPhen [8], and VARITY [16]. The importance of conservation is expected and indicates the validity of MAGPIE’s learned representations. Additionally, we observed that features within each modality are correlated, while features from different modality groups are more likely to be independent (Fig. 2A). This suggests that MAGPIE is capable of learning multiple-dimensional information from different feature classes. In other words, the learned representations were discriminative to help MAGPIE for classification.

### MAGPIE outperforms existing methods across multiple conditions

**Fig. 3:**
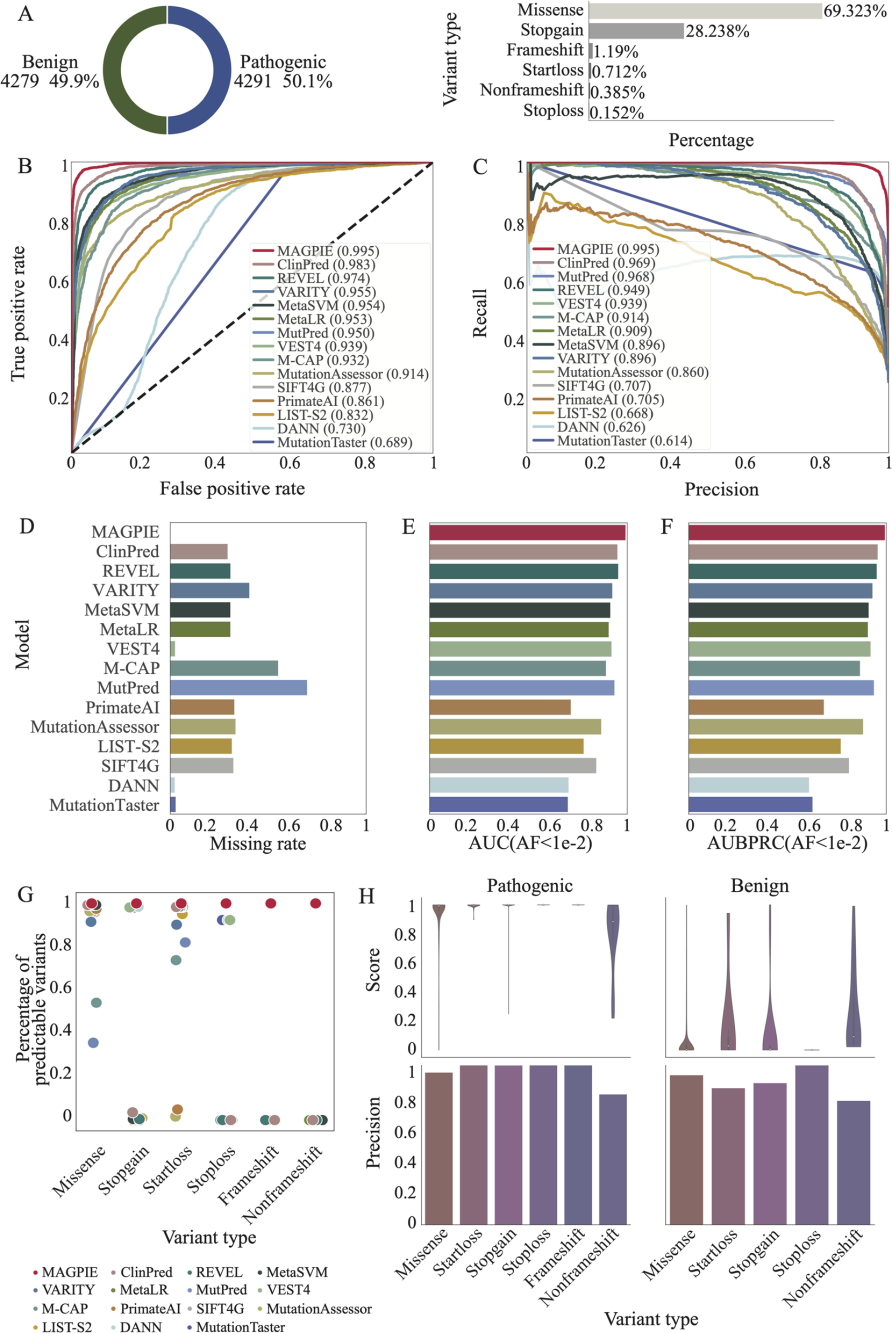
MAGPIE makes accurate predictions. **A** The pie chart showed the proportion of pathogenic and benign variants in the independent test set, and the bar plot illustrated the percentages of multi-type variants in the dataset. **B** The receiver operating characteristic curve of MAGPIE and 14 other predicted tools in the independent test set. The area under the curve (AUC) scores were shown in the bar plot. **C** Precision-recall curve of MAGPIE and 14 other predicted tools in the ClinVarTest dataset were illustrated. **D** Missing rate comparison of MAGPIE and 14 other predicted tools in the independent test set. The higher missing rate represented that the prediction tools cannot predict pathogenic scores on the larger number of candidate variants. **E** AUC comparison of MAGPIE and 14 other predicted tools in the ClinVarRare, which only included variants with AF < 0.01. **F** AUBPRC comparison of MAGPIE and 14 other predicted tools in the ClinVarRare which only included variants with AF < 0.01. **G** Percentages of predictable variants across different variant types in various tools. **H** Violin plots illustrated distributions of pathogenic scores. And bar plots showed the precisions in each category of pathogenic variants and benign variants

We evaluated the performance of MAGPIE together with 14 previously published pathogenicity prediction methods including MutationAssessor, MetaSVM, MetaLR, VARITY, VEST4, REVEL, MutPred, DANN, ClinPred, PrimateAI, LIST-S2, M-CAP, MutationTaster, and SIFT4G [11, 13, 14, 16, 17, 21, 22, 31–36]. For benchmark purposes, we applied MAGPIE on the ClinVar training dataset as described in methods, and its independent split dataset, labeled as ClinVarTest hereafter, was used for evaluating all models, including MAGPIE performances (Fig. 3A). The ClinVarTest dataset contains 4356 pathogenic and 4310 benign mutations. Since we split datasets based on gene symbols, these variants and their corresponding gene-level features were not seen by the models in the training dataset (Fig. 3A, Additional file 1: Table S1). To fairly compare MAGPIE with other methods, pathogenicity classifications were set according to the thresholds recommended by the authors. Nevertheless, MAGPIE outperformed all other classifiers on the benchmark with the highest AUC score of 0.995 and AUPRC of 0.995 (Fig. 3B, C, Additional file 1: Table S6-S8). In comparison, other methods, i.e., MutationTaster, DANN, LIST-S2, SIFT4G, PrimateAI, M-CAP, and MutationAssessor achieved AUC from 0.61 to 0.91. The performance evaluation suggested that MAGPIE is a reliable tool for pathogenic prediction.

Of note, existing pathogenic prediction tools mostly predicted a limited number of mutations, which indicated a lack of generalization of previous methods. For example, for published classifiers, i.e., ClinPred, REVEL, MetaSVM, MetaLR, MutationAssessor, PrimateAI, SIFT4G, LIST-S2, and M-CAP, 30–60% of candidate mutations were unpredictable by these methods (Fig. 3D), which leads to 30–60% of variants’ output as missing. These missing values were generated due to variant types not being predictable by specific prediction tools. In contrast, MAGPIE succeeded in predicting the pathogenicity of all variants in the dataset, fulfilling 3–60% of missing values predicted by other methods (Fig. 3C). In other words, for all the exonic variants assessed covering various mutation types other than synonymous mutation, the missing rate of MAGPIE is zero. In summary, MAGPIE outperformed all previously published machine learning methods as well as the state-of-the-art deep learning methods on this benchmark.

Finally, we constructed a rare mutation test set (ClinVarRare) to evaluate the ability of MAGPIE to identify pathogenic variants from rare benign variants, which is one of the major challenges in real-world analysis for genetic diagnosis of disease. The ClinVarRare test set included rare pathogenic ClinVar variants (AF < 0.01) and rare benign variants from gnomAD (AF < 0.01), which is more similar to the real whole-exome sequencing datasets. For the rare mutation test set, MAGPIE achieved the best performance with an AUC equal to 0.992, followed by REVEL, ClinPred, and MutPred. Previous computational tools predicted a proportion of variants within the whole dataset, and the rest of the variants were unpredictable because these tools were designed to classify specific types of variants. Consequently, we examined that MAGPIE predicted all 4881 variants in the ClinVarRare test set with the highest accuracy of 0.95 (Fig. 3E). In summary, MAGPIE outperforms all previously published machine learning methods and the state-of-the-art deep learning methods on both the ClinVarTest dataset and the ClinVarRare test dataset.

### MAGPIE achieves high performance in multiple mutation types

An important characteristic of MAGPIE is the ability to make pathogenic predictions on multiple types of mutations. The ClinVarTest dataset contains a total of 6 types of mutations: missense variants (or nonsynonymous SNV), stop-gain variants, start-loss variants, frameshift mutations, nonframeshift mutations, and stop-loss variants. MAGPIE was predictive of clinical significance for all labeled variants across various mutation types, while many available methods only applied to certain mutation types (Fig. 3G). Besides, the performance of our methods was robust to different variant types, suggesting the generalizability of our method (Fig. 3H).

We further analyzed the distribution of pathogenic scores across mutation types to evaluate the classification capability of MAGPIE. To visualize the distribution of MAGPIE-predicted pathogenic probability, we plotted the MAGPIE score distributions for different types of pathogenic and benign variants (Fig. 3H). For pathogenic variants, MAGPIE scores were highly concentrated around 1, and for benign variants, scores were near 0 across six types of variants. This suggested that our model can separate pathogenic and benign variants well. We further analyzed the ability of classification quantitatively and evaluated how accurately our model can predict pathogenicity. The average precision of all types of variants was 0.98, suggesting that 98% of predicted pathogenic variants were consistent with their true labels. And the average false predictive value was 0.8, meaning 80% of predicted benign mutations were true negatives. In particular, MAGPIE identified pathogenic and benign variants successfully in the imbalanced datasets of stop-loss and stop-gain mutations. As shown in Fig. 3G, MAGPIE detected pathogenic mutations in stop-loss and stop-gain mutations and minimized false positives (average precision of 1.0). Therefore, MAGPIE can accurately differentiate pathogenic and benign mutations across various mutation types.

### MAGPIE achieves the best performance on additional orthogonal datasets

**Fig. 4:**
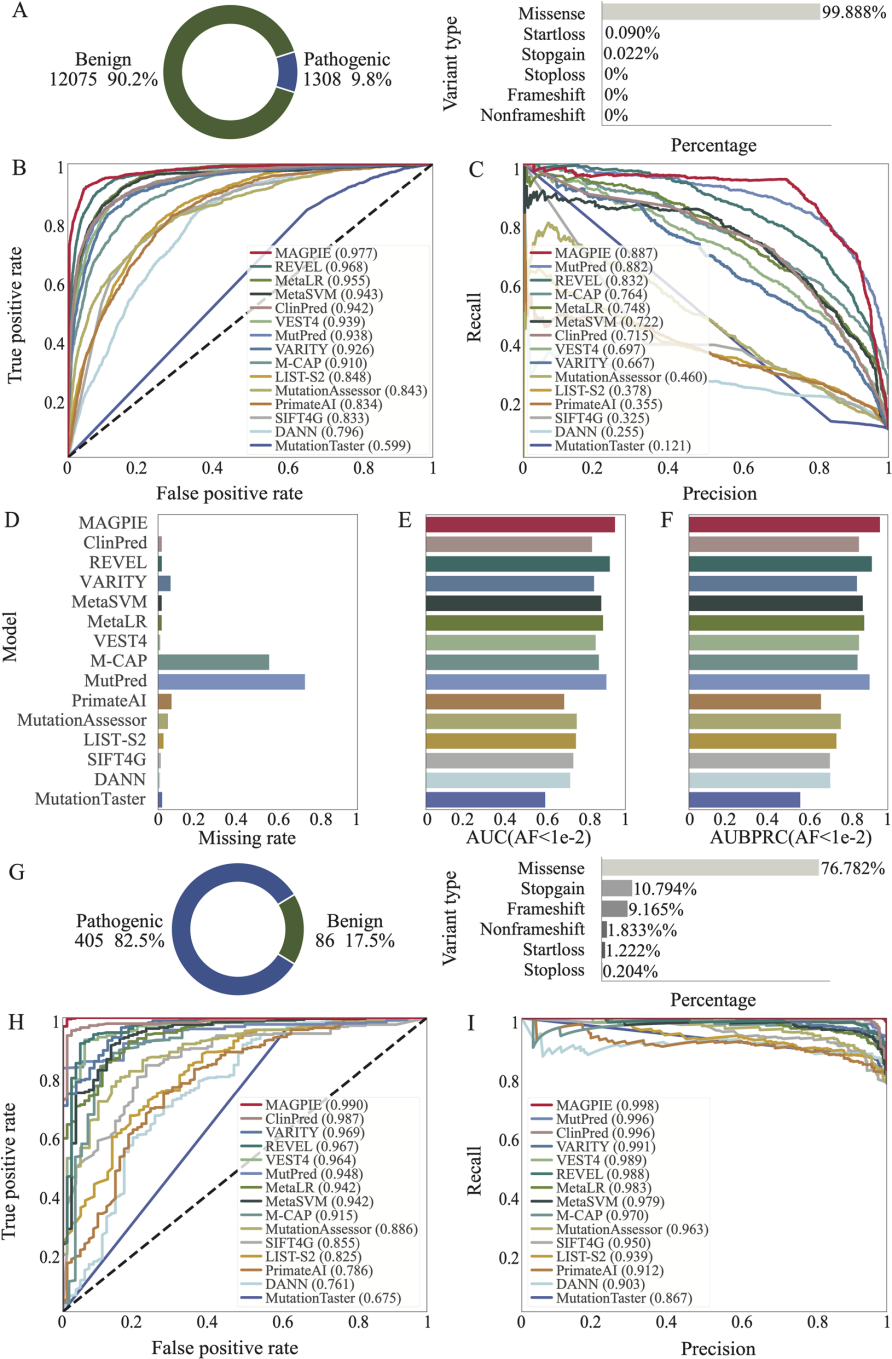
MAGPIE outperforms other models in orthogonal validation set and ACMG-guided dataset. **A** The pie chart showed the proportion of pathogenic and benign variants in the orthogonal validation set and the bar plot illustrated the percentages of multi-type variants in the dataset. **B** The receiver operating characteristic curve of MAGPIE and 14 other predicted tools in the orthogonal validation set. **C** The precision-recall curve of MAGPIE and 14 other predicted tools in the orthogonal validation set were illustrated. **D** Missing rate comparison of MAGPIE and 14 other predicted tools in the orthogonal validation set. The higher missing rate represented that the prediction tools cannot predict pathogenic scores on the larger number of candidate variants. **E** AUC comparison of MAGPIE and 14 other predicted tools in the SwissProtRare which only included variants with AF < 0.01. **F** AUBPRC comparison of MAGPIE and 14 other predicted tools in the SwissProtRare which only included variants with AF < 0.01. **G** The pie chart showed the proportion of pathogenic and benign variants in the ACMG-guided dataset, and the bar plot illustrated the percentages of multi-type variants in the dataset. **H** Performance comparison of MAGPIE and 14 other predicted tools in the ACMG-guided dataset. **I** The precision-recall curve of MAGPIE and 14 other predicted tools in the ACMG-guided dataset were illustrated

We used the annotated variants from the SwissProt database as an orthogonal dataset to further benchmark MAGPIE against other methods. SwissProt contains 80,840 labeled variants, with 71,400 explicitly labeled as pathogenic or benign. Only variants with unique reference SNP ID mapping, in the exonic region, excluding synonymous SNVs, were retained. To ensure the independence of the orthogonal dataset, we further removed variants located on genes reported in the ClinVarTrain dataset. We also made the orthogonal dataset closer to the real scenario and evaluate the performance of MAGPIE in the imbalanced dataset with less than 10% positive samples. After filtering, the number of pathogenic and benign mutations became highly imbalanced. The number of benign variants was 12,075, while the number of pathogenic ones was only 1308, about 9.8% of the total number of variants to be tested (Fig. 4A, Additional file 1: Table S2). We also conducted an ablation study in this imbalanced orthogonal dataset and found that the full model was much superior to other model variations of MAGPIE (Additional file 2: Fig. S2).

Again, the performance of MAGPIE was superior to other machine learning and deep learning methods on this benchmark. MAGPIE outcompeted other tools with the best AUC of 0.97 and AUPRC of 0.88 (Fig. 4B, Additional file 1: Table S6-S8). Furthermore, MAGPIE computed the pathogenic probability for all filtered variants without missing values (Fig. 4C), whereas VEST4, DANN, MutationTaster, and MetaSVM were the closest competitor with about 5% missing rate (Fig. 4C, Additional file 1: Table S6-S8). As for M-CAP and MutPred, more than 40% variants were unpredictable. Hence, on this benchmark, MAGPIE was better than other methods in predicting novel pathogenic mutations.

To evaluate the performance of MAGPIE in the imbalanced test dataset with rare variants, we constructed a rare SwissProt test set (SwissProtRare) where all variants had AF less than 0.01. The dataset included 9% of rare pathogenic SwissProt variants and 91% of rare benign variants from gnomAD, an extreme case of an imbalanced rare variant dataset. MAGPIE outperformed all competitors with an AUC equal to 0.95, followed by REVEL (AUC = 0.92) and MutPred (AUC = 0.91) (Fig. 4E). In general, MAGPIE outperformed other competitors.

Three types of variants were included in the SwissProt dataset, and MAGPIE was able to differentiate well between pathogenic and benign variants across all variant types in the orthogonal dataset as well (Additional file 2: Fig. S4A). Additionally, we found that MAGPIE scores of pathogenic variants were concentrated close to 1. In contrast, the probabilities of benign variants were near 0, which will further support the reliability of our method (Additional file 2: Fig. S4B). Precision of pathogenic variants was higher than 0.85, suggesting that MAGPIE achieved accurate pathogenic variants prediction (Additional file 2: Fig. S4B). Consistent with the results in the ClinVarTest dataset, for start-loss and stop-gain variants, MAGPIE achieved higher precision than the precision of nonsynonymous SNVs (precisions of 0.95 and 1, respectively) (Additional file 2: Fig. S4B). The false predictive values of stop-loss and stop-gain variants were slightly lower than the false predictive values of nonsynonymous SNVs and start-loss variants. However, the average false predictive value achieved 0.8 (Additional file 2: Fig. S4B). Thus, MAGPIE could successfully predict new pathogenic variants among different variant types.

Furthermore, we assessed the performance of MAGPIE and other models on an additional validation set from ACMG test panel. We obtained the ACMG guided test panel (2023.05.22), which includes a total of 1270 mutations, from the official website. After filtering, a final dataset of 491 mutations was used to compare MAGPIE and other model performances. In contrast to the SwissProt dataset, in the imbalanced ACMG dataset, there were 82% of pathogenic variants and 18% of benign variants. Again, MAGPIE achieved the highest AUC and AUPRC among all methods (Additional file 2: Fig. S5-S6, Additional file 1: Table S6-S8), illustrating that MAGPIE could classify pathogenic and benign in imbalanced datasets.

### Assessing MAGPIE performance with varying threshold

Different thresholds may affect the model performance, especially in imbalanced data. In the real-world clinical applications of pathogenic prediction tools to interpret VUS in individual patients, only a few pathogenic mutations should be identified out of tens of thousands of candidate variants called from WES data. Thus, the pathogenic prediction tasks are presumably assigned to handle highly imbalanced datasets. Therefore, we investigated the optimal threshold of MAGPIE under various data balance scenarios. The default parameter of our model is 0.5 in balanced datasets such as the ClinVarTest dataset. We found that with the predicted probabilities between 0.4 and 0.7, both the accuracy and F1-score were stable (Additional file 2: Fig. S7-S8). Besides, Matthew’s correlation coefficient (MCC), a measure of association for two binary variables, in the ClinVarTest dataset confirmed that 0.5 was a reliable threshold for this benchmark (Additional file 2: Fig. S7-S8).

To examine the effects of different ratios between the number of pathogenic and benign variants, the ClinVarTest dataset was randomly divided into a series of subsets with various combinations. We randomly added different proportions of pathogenic variants to all benign variants from the ClinVarTest dataset to construct independent test subsets. We can see that in such cases, the optimal thresholds had dropped from 0.95 to 0.72 (Additional file 2: Fig. S7). However, MCC, accuracy, and F1-score were relatively stable in these subsets, which illustrated that performances of MAGPIE had less impact caused by the different thresholds (Additional file 2: Fig. S7). Furthermore, we added various percentages of benign variants to all pathogenic variants. We also found similar results (Additional file 2: Fig. S8).

We next studied the orthogonal SwissProt dataset, which was highly imbalanced with only 9.8% pathogenic mutations, and the ACMG dataset, which only included 18% benign variants. The SwissProt dataset is more similar to real applied scenarios than the ClinVarTest dataset and has clear labels of pathogenicity. Therefore, a precision-recall curve can help us to evaluate the influence of classifier performance with different thresholds. We found that the optimal threshold in this dataset was 0.71 (Additional file 2: Fig. S9A). We analyzed the performance matrix in the dataset with the default threshold and the optimal threshold. Our findings showed that the performance of MAGPIE became better with stricter thresholds. MCC and F1-score increased by 2% and 3%, respectively (Additional file 2: Fig. S9A). Similarly, we observed that the optimal threshold of the ACMG dataset was 0.34 (Additional file 2: Fig. S9B). MCC and F1-score had been slightly improved. However, if there is no precise estimate of the compositions of the dataset, MAGPIE with the default parameter still outperformed other methods. Therefore, using a default threshold can also achieve accurate prediction on both imbalanced and balanced datasets as shown before.

### MAGPIE prediction on gene mutations that cause Mendelian diseases

**Fig. 5:**
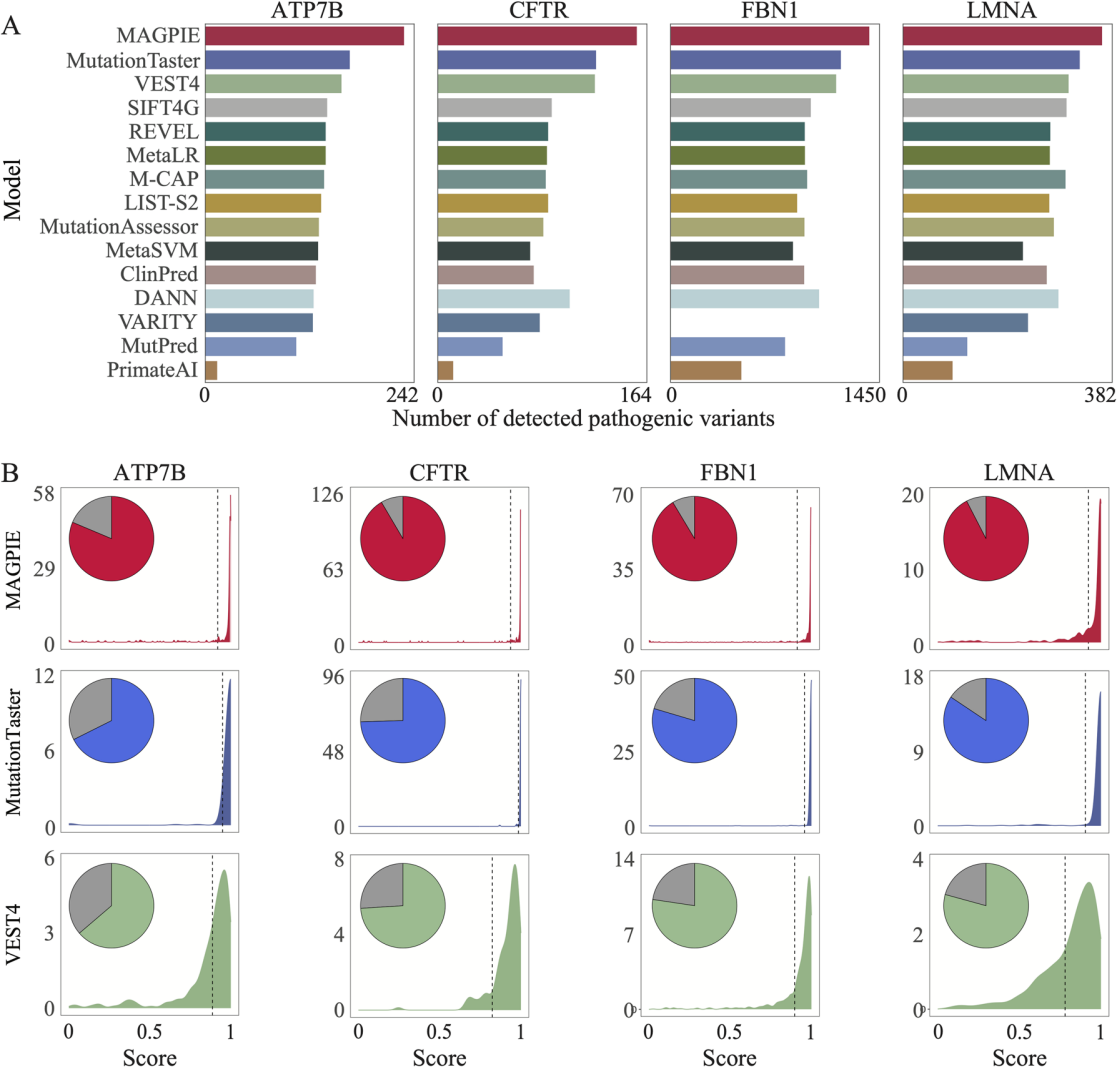
MAGPIE detects most variants in pathogenic genes. **A** Comparison of the number of detected pathogenic variants in four well-known pathogenic genes between MAGPIE and 14 other prediction tools. **B** Density plots illustrated distributions of pathogenic scores predicted by MAGPIE, MutationTaster, and VEST4. Moreover, the pie charts showed the proportion of predictable and unpredictable variants of each tool

Next, we applied MAGPIE to four genes curated with a large number of known pathogenic mutations causing different Mendelian diseases (Methods). For all the four genes tested, i.e., ATP7B, CFTR, FBN1, and LMNA, MAGPIE was able to recover the largest number of known pathogenic mutations compared to other methods with default parameters (Fig. 5A). On average, MAGPIE recovered 96% of known pathogenic variants. For instance, for FBN1, the gene mutated in Marfan syndrome [39] and included 1621 pathogenic mutations. MAGPIE predicted 1450 (87%) candidate variants as pathogenic, and MAGPIE scores of 95% pathogenic variants were close to 1 (Fig. 5B). All other tools correctly classified a smaller fraction of variants, which was lower than 70%. For CFTR (which is associated with cystic fibrosis), MAGPIE predicted 164 (95%) variants to be pathogenic. Compared to MutationTaster, VEST4, and other tools, MAGPIE scores were concentrated about 1 (Fig. 5B). Moreover, the performance of our classifier on ATP7B and FBN1 was better than other methods, indicating a stable performance for evaluating pathogenicity despite using entirely different disease-associated genes (Fig. 5A, B). Thus, MAGPIE performs better in identifying pathogenic mutations that cause Mendelian diseases.

## Discussion

The next-generation sequencing has revealed tremendous amount of genetic variations in the human population. However, it has raised another challenge: the difficulty of identifying the actual disease-causing mutation from the millions of normal variations. The current computation tools have enabled to predict one or several specific types of mutations. For example, M-CAP, MutationTaster, and PrimateAI focus on missense variants. However, specific types of variants have been filtered out due to the models’ scope. Various types of mutations present the challenge of the generalization ability for computational methods to predict pathogenic scores among mutations. Therefore, it is critical to train a generalized classifier foremost to expand predictable variant types.

To address this challenge, we report a computational classifier named MAGPIE, which focuses on classifying multiple types of mutations of uncertain significance for Mendelian diseases. The novelty of this work comes from a combination of data preparation, feature extraction, feature engineering, and model selection. The overall idea is that from the users’ perspective, the pathogenicity of a variant should be jointly evaluated on multiple scopes, which corresponds to features derived from six modalities in MAGPIE. Therefore, the machine learning components of MAGPIE are designed to maximize the models’ learning on multimodal data.

The machine learning component of MAGPIE mainly included autoFE and LightGBM, both of which we think contributes to the novelty of the method. In the pathogenicity prediction task, the pathogenicity of variants may be caused by multiple types of features. To enhance the model’s performance, we aimed to integrate these multimodal features. By incorporating the autoFE component, MAGPIE has the capability to handle the complexity of the features and effectively capture the underlying relationships. This enables us to extract valuable information from the features and improve the overall performance of our model. This process involves applying certain transformations to the features, allowing for a better representation of their performance in predicting mutation pathogenicity, such as through Group-By operations. Of note, MAGPIE employs separated feature selection and step-wise training approaches during the autoFE process to avoid overfitting. In our updated ablation study, the inclusion of the autoFE component enhances the performance of MAGPIE (Additional file 2: Fig. S2).

The input data for pathogenicity prediction in MAGPIE is in tabular format, which makes it suitable for tree-based models. Among the tree-based models, LightGBM stands out as one of the most efficient models in terms of both prediction accuracy and computational time. LightGBM is based on the gradient boosting framework and incorporates a number of advanced techniques such as histogram-based binning, leaf-wise tree growth, and gradient-based one-side sampling. These techniques improve model performance and enables faster training and inference times compared to other tree-based models or machine learning models. Consistently, in the ablation study for MAGPIE, we found that the computational time required for SVM with an identical training process was approximately 10 times longer than LightGBM. By incorporating LightGBM in MAGPIE, we are able to leverage its efficiency and accuracy to achieve accurate and timely pathogenicity predictions (Additional file 2: Fig. S10). Moreover, we can easily calculate feature importance from the LightGBM model, which enables the interpretability of MAGPIE.

Another novel aspect of MAGPIE design is the ability to make accurate prediction for rare variants, which is highly clinically relevant but not naturally well represented in ClinVar database as training source. In real-world pathogenicity prediction and diagnosis scenarios, it is common to encounter rare benign variants. Therefore, we aimed to include rare benign variants in the training of MAGPIE to enhance its ability to detect pathogenic variants in clinical settings. However, in large mutation datasets like ClinVar, rare benign variants make up a very small proportion of the dataset. To address this issue, we incorporated rare benign variants from gnomAD into the training set. By including rare benign variants from gnomAD, we were able to overcome the lack of features associated with rare benign variants in ClinVar. This inclusion of a more diverse dataset, encompassing rare benign variants, led to better generalization of the MAGPIE model. Ablation study also proves this crucial role of relevant cross-dataset working in improving model performance (Additional file 2: Fig. S2). In the ablation study, we find that removing the data from gnomAD in the training set has a significant impact on the performance of the MAGPIE model. Particularly in the pathogenicity prediction task for rare mutations, adding gnomAD mutations to the training set significantly increases the probability of correctly classifying rare benign mutations (Additional file 2: Fig. S2).

On the ClinVarTest dataset and the orthogonal datasets, our approach accurately classified multi-type variants, respectively, whereas most tools can only predict one or two types of variants. However, the total numbers of variants across variant types in the ClinVar dataset were different. Compared with the number of missense variants, other types of variants, such as stopgain, stoploss, and so on, had less number of records. Thus, the small sample size in these variant types might limit, to some extent, the performance of MAGPIE and cause some biases.

Many previous studies have demonstrated outstanding performance in accurately predicting pathogenic mutations and have paved the way for subsequent work. The design of MAGPIE draws inspiration from many previous work, such as utilizing the AUBPRC metric defined in VARITY to assess the performance of imbalanced datasets. However, we showed here that the state-of-the-art methods included missing value because their predictions are concentrated on one or several specific mutation types. In the clinical application for genetic diagnosis, these unpredictable variants would be filtered out and often be treated as non-pathogenic ones (Additional file 2: Fig. S11).

Detection of pathogenic variants using MAGPIE may suffer from several limitations. First, we evaluated the running time of our method and found that the annotation tools cost the most time in the MAGPIE pipeline since the running time of MAGPIE was limited by the necessary file I/O in annotation tools (Additional file 2: Fig. S11). Also, our approach does not address the influences of a combination of variants. Human diseases may be associated with multiple causative mutations. However, our method focuses on predicting the pathogenicity of a single variant. Another potential limitation is that this approach is designed to obtain information from genetic annotations without any phenotype-related annotations. Future methods development may consider features including phenotype-related annotations to build a more direct connection between diseases and genetic variants. Currently, the clinical work of genetic diagnosis rate is still limited. It was reported that using WES data, the genetic diagnosis rate can be up to 40%, suggesting a large space of improvement remains for practical clinical work. Several reasons lead to this result, one of which is that most of the human genome is non-coding, while most algorithms can only predict a subset of the coding region. Admittedly, computational predictions focusing on exonic mutations other than synonymous mutations like MAGPIE are necessary but still insufficient to address all the clinical diagnosis challenges. MAGPIE cannot provide reliable predictions for intronic and intergenic variants due to the scarcity of annotation information in existing databases and the lack of training sources. As we move forward, we will endeavor to acquire essential information about these types of mutations.

## Conclusions

In this study, we introduce a computational framework named MAGPIE, which generates pathogenicity scores for multi-type variants and simplifies pathogenicity classification for millions of candidate genomic variants. Moreover, MAGPIE is independent of any other pathogenic prediction tools, thereby avoiding introducing biases and circularity. Our approach yields superior performance and low missing rates for various types of exonic variants in both balanced and imbalanced datasets. Besides, MAGPIE also provided an accurate prediction of multi-type rare variants. Furthermore, MAGPIE performance remained robust, recovering known pathogenic variants across different Mendelian diseases. In conclusion, with improved prediction accuracy, MAGPIE provides a more accessible and accurate prediction of multi-type exonic variants for Mendelian disease studies.

### Supplementary Information

**Additional file 1: Table S1.** Category and proportion of variants in independent test set. **Table S2.** Category and proportion of variants in orthogonal validation set. **Table S3.** Category and proportion of variants in ACMG guided dataset. **Table S4.** Feature name and description. **Table S5.** Feature importance of MAGPIE. **Table S6.** Performance metrics in independent test set. **Table S7.** Performance metrics in orthogonal validation set. **Table S8.** Performance metrics in ACMG guided dataset.

**Additional file 2: Fig. S1.** Web tool of MAGPIE. **Fig. S2.** Ablation study of MAGPIE. **Fig. S3.** Significance test on independent test set. **Fig. S4.** Performance of each variant type on orthogonal validation set. **Fig. S5.** Performance evaluation on ACMG guided dataset. **Fig. S6.** Performance of each variant type on ACMG guided dataset. **Fig. S7.** Threshold test of MAGPIE on independent test set when number of pathogenic variants is less than benign ones. **Fig. S8.** Threshold test of MAGPIE on orthogonal validation set when the number of pathogenic variants is less than benign. **Fig. S9.** Performance comparison before and after threshold adjustment according to FPR control. **Fig. S10.** MAGPIE running time. **Fig. S11.** Performance comparison in real-world diagnosis.

## Data Availability

Datasets generated and/or analyzed during the current study are publicly available in the ClinVar (https://ftp.ncbi.nlm.nih.gov/pub/clinvar/) [4], SwissProt (https://ftp.uniprot.org/pub/databases/uniprot/) [40], gnomAD (https://gnomad.broadinstitute.org/downloads/) [1], and ACMG guided dataset (10.1016/j.gim.2021.11.018) [41]. The datasets supporting the conclusions of this article are included within the article and its additional files. MAGPIE web-based prediction tool for all nonsynonymous exonic variants is available at http://tools.shenlab-genomics.org/tools/MAGPIE.

## Abbreviations

MAGPIE: Multimodal Annotation Generated Pathogenic Impact Evaluator
ClinVar: Clinical variation
AUC: Area under curve
WGS: Whole genome sequencing
WES: Whole exome sequencing
gnomAD: Genome Aggregation Database
ExAC: Exome Aggregation Consortium
ML: Machine learning
DL: Deep learning
OMIM: Online Mendelian Inheritance in Man
HGMD: Human Gene Mutation Database
RNA: Ribonucleic acid
SpliceAI: Splice artificial intelligence
EVE: Evolutionary model of variant effect
CADD: Combined Annotation-Dependent Depletion
DANN: Deleterious Annotation of Genetic Variants using Neural Networks
REVEL: Rare Exome Variant Ensemble Learner
MutPred: Mutation predictor
VEST: Variant effect scoring tool
PROVEAN: Protein Variation Effect Analyzer
PhastCons: Phylogenetic Analysis with Space/Time Models for Conservation of Evolutionary Significant Sequences
GBM: Gradient-boosting model
GOSS: Gradient-based one-side sampling
EFB: Exclusive feature bundling
GDI: Gene damage index
LoF: Loss of function
SIFT: Sorting Intolerant From Tolerant
PolyPhen: Polymorphism phenotyping
MetaSVM: Meta-learner support vector machine
MetaLR: Meta-logistic regression
ClinPred: Clinical variant prediction
PrimateAI: Primate artificial intelligence
LIST-S2: Local Identity and Shared Taxa—S2
M-CAP: Mendelian Clinically Applicable Pathogenicity
SIFT4G: Sorting Intolerant From Tolerant for Genomes
SNV: Single-nucleotide variant
AUPRC: Area under the precision-recall curve
AUBPRC: Area under the balanced precision-recall curve
MCC: Matthews correlation coefficient
GWAS: Genome-wide association study
ANNOVAR: Annotation of variants from next-generation sequencing data using a reference genome
RVIS: Residual variation intolerance score
LoFtool: Loss of function tool
DS: Delta score
AG: Acceptor gain
AL: Acceptor loss
DG: Donor gain
DL: Donor loss
DP: Delta position
AF: Allele frequency
phyloP: Phylogenetic *P*-values
SiPhy: SIte-specific PHYlogenetic analysis
AutoFE: Automatic feature engineering
SFS: Separated feature selection
ROC: Receiver operating characteristic
