# Supplementary material for: MAGPIE: accurate pathogenic prediction for multiple variant types using machine learning approach
Source: Genome Med. 2024 Jan 8;16:3. doi: 10.1186/s13073-023-01274-4 (PMC10773112; doi:10.1186/s13073-023-01274-4)
Supplement: Supplementary file 2 — Additional file 2: Fig. S1. Web tool of MAGPIE. Fig. S2. Ablation study of MAGPIE. Fig. S3. Significance test on independent test set. Fig. S4. Performance of each variant type on orthogonal validation set. Fig. S5. Performance evaluation on ACMG guided dataset. Fig. S6. Performance of each variant type on ACMG guided dataset. Fig. S7. Threshold test of MAGPIE on independent test set when number of pathogenic variants is less than benign ones. Fig. S8. Threshold test of MAGPIE on orthogonal validation set when the number of pathogenic variants is less than benign. Fig. S9. Performance comparison before and after threshold adjustment according to FPR control. Fig. S10. MAGPIE running time. Fig. S11. Performance comparison in real-world diagnosis. [file 13073_2023_1274_MOESM2_ESM.pdf]

Supplementary Figures

MAGPIE: accurate pathogenic prediction for multiple variant types using machine learning approach

Fig. S1

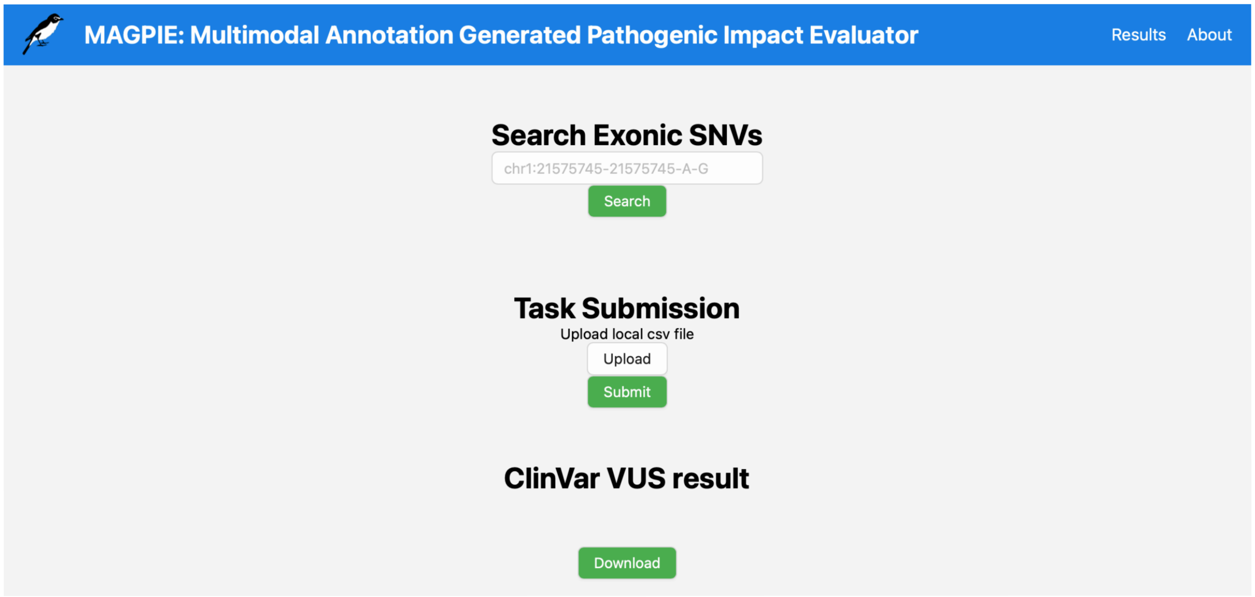

Web tool of MAGPIE. Users of the MAGPIE tool can effortlessly obtain pathogenicity scores for exonic Single Nucleotide Variants (SNVs) by searching for the web page. Furthermore, users can upload local vcf files to initiate predictions for non-SNVs or SNVs currently not included in the system.

Fig. S2

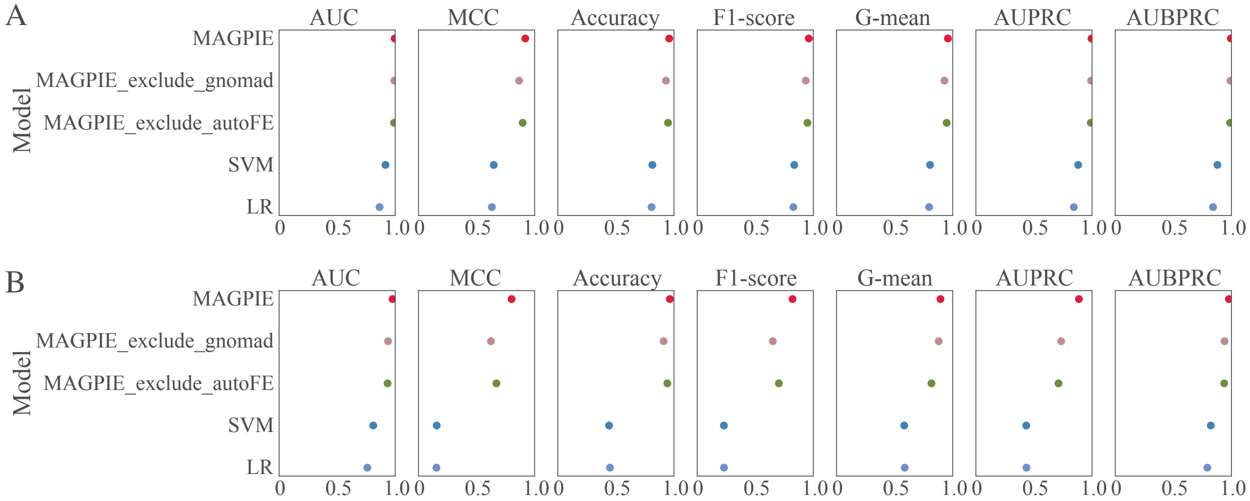

Ablation study of MAGPIE. A Metrics used to evaluate the performance of ablation study on independent test set. **B** Metrics used to evaluate the performance of ablation study on orthogonal validation set.

Fig. S3

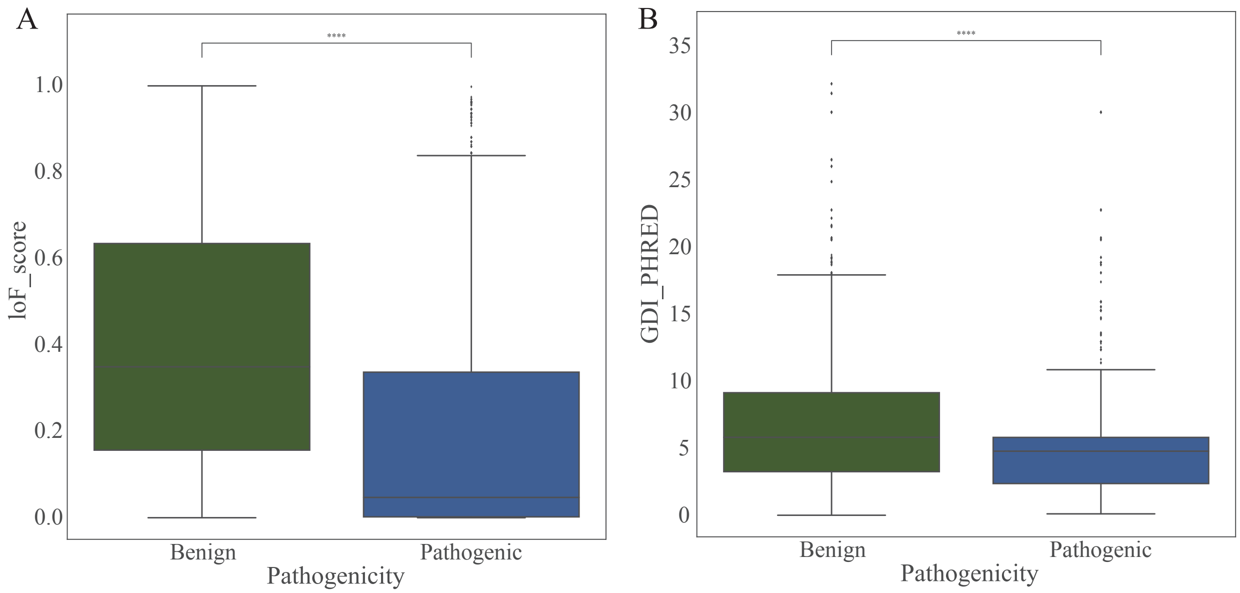

Significance test on independent test set. **A** LoF_score significantly decreases in pathogenic variants compared to benign variants. **B** GDI significantly decreases in pathogenic variants compared to benign variants.

Fig. S4

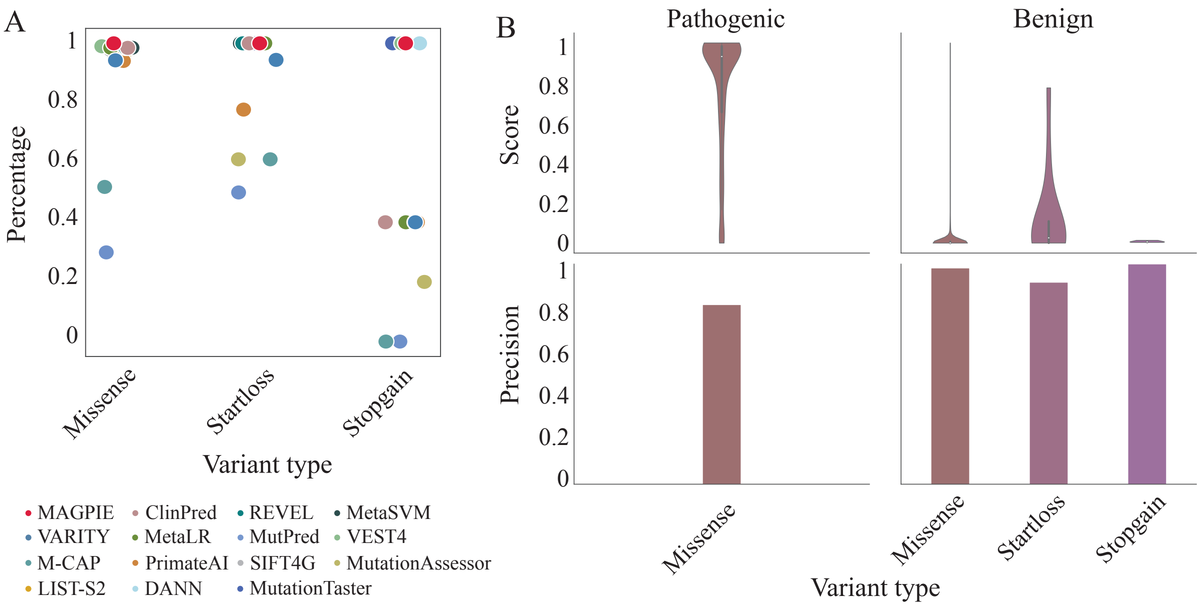

Performance of each variant type on orthogonal validation set. A Percentages of predictable variants across different variant types in various tools. **B** Violin plots illustrated distributions of pathogenic scores. And bar plots showed the precisions in each category of pathogenic variants and benign variants.

Fig. S5

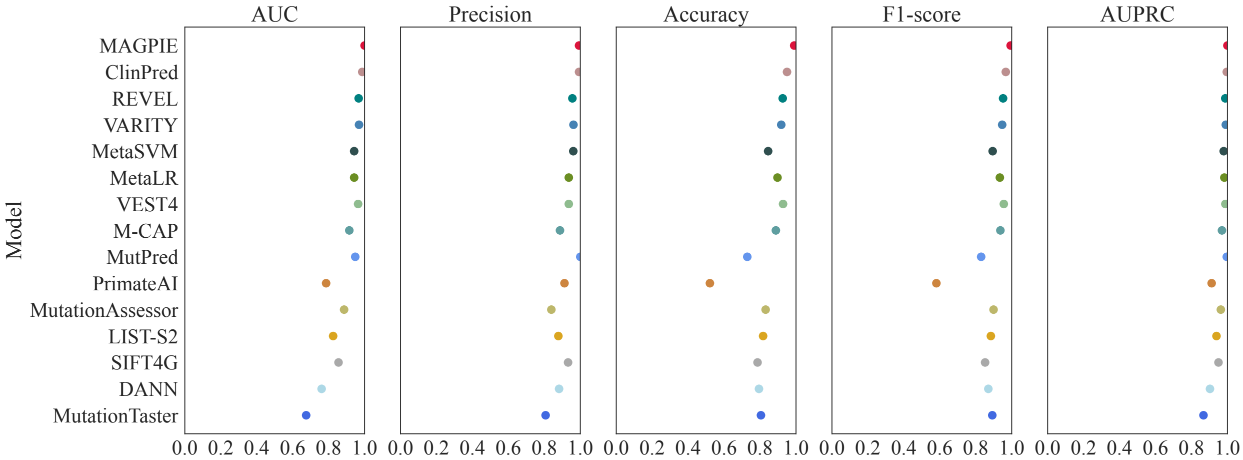

Performance evaluation on ACMG guided dataset. MAGPIE can outperform other prediction tools on ACMG-guided dataset.

Fig. S6

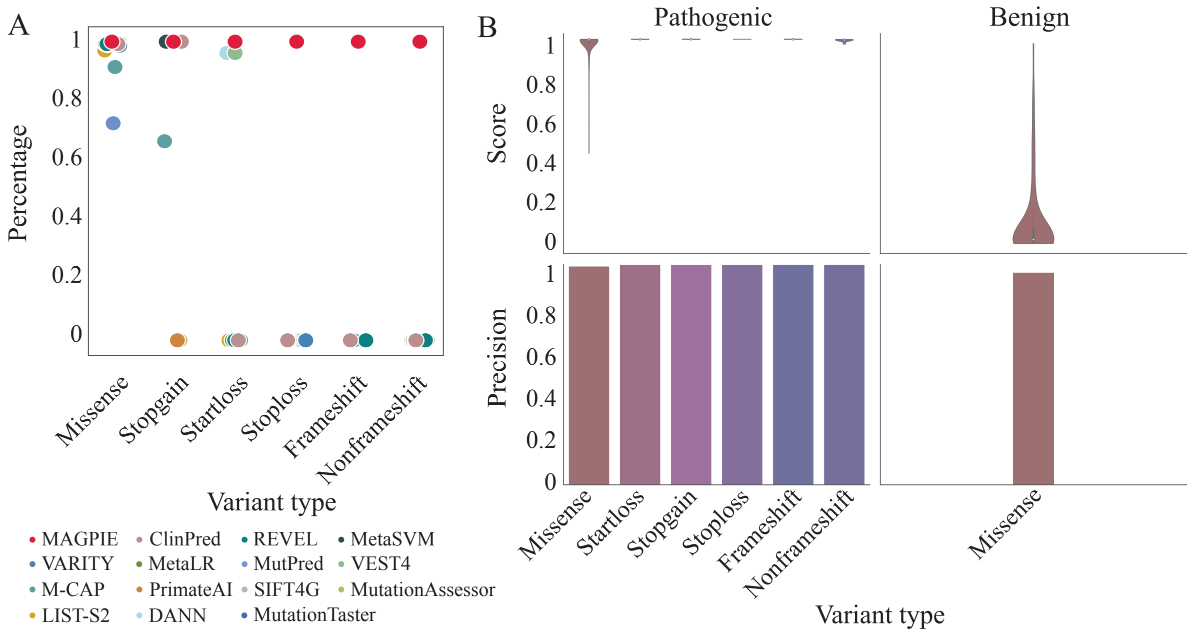

Performance of each variant type on ACMG guided dataset. **A** Percentages of predictable variants across different variant types in various tools. **B** Violin plots illustrated distributions of pathogenic scores. And bar plots showed the precisions in each category of pathogenic variants and benign variants.

Fig. S7

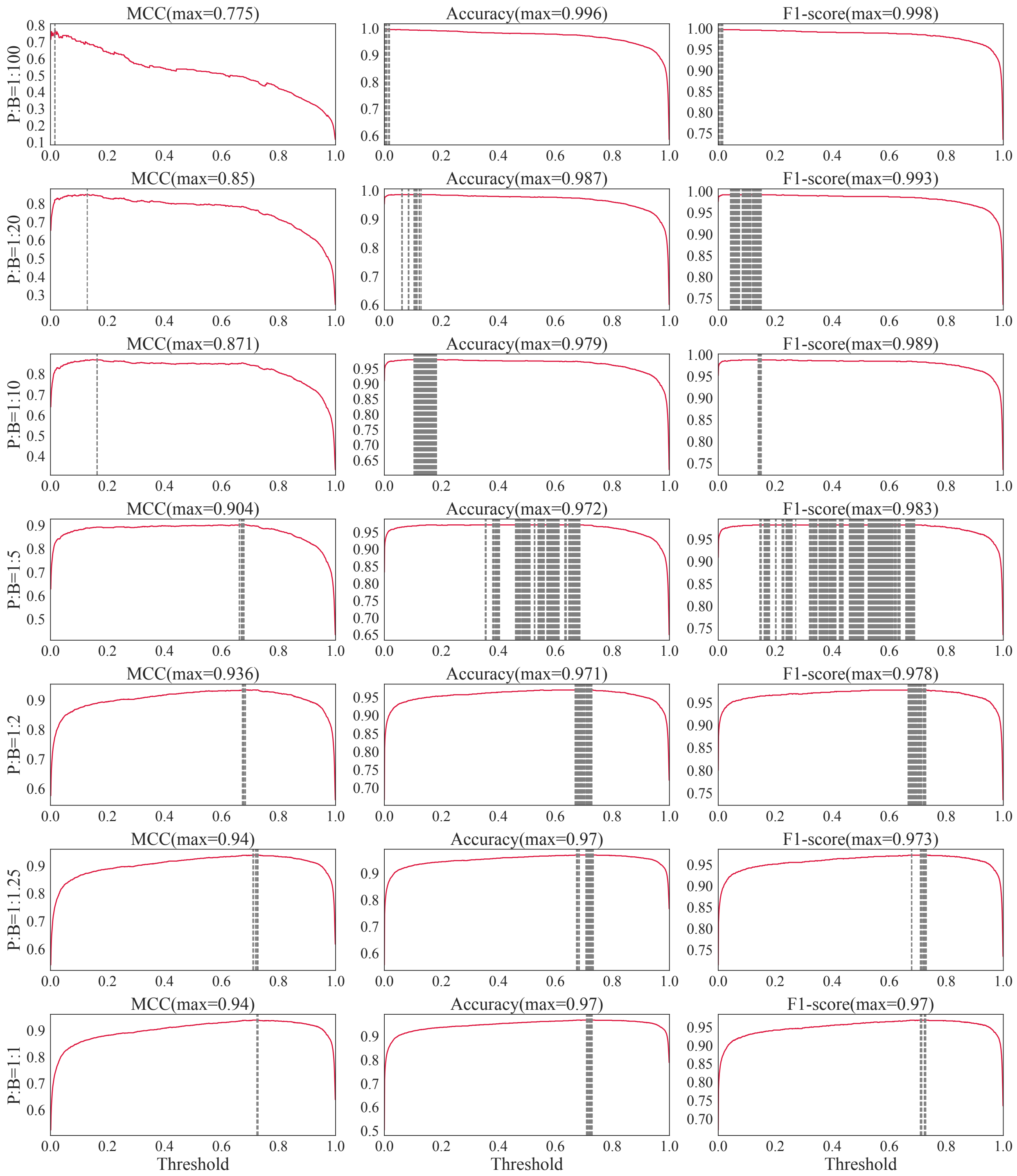

Threshold test of MAGPIE on independent test set when number of pathogenic variants is less than benign ones. We set different ratios of pathogenic variants to make balanced and imbalanced datasets and plot the performance metrics over changes. B and P stand for benign and pathogenic, respectively.

Fig. S8

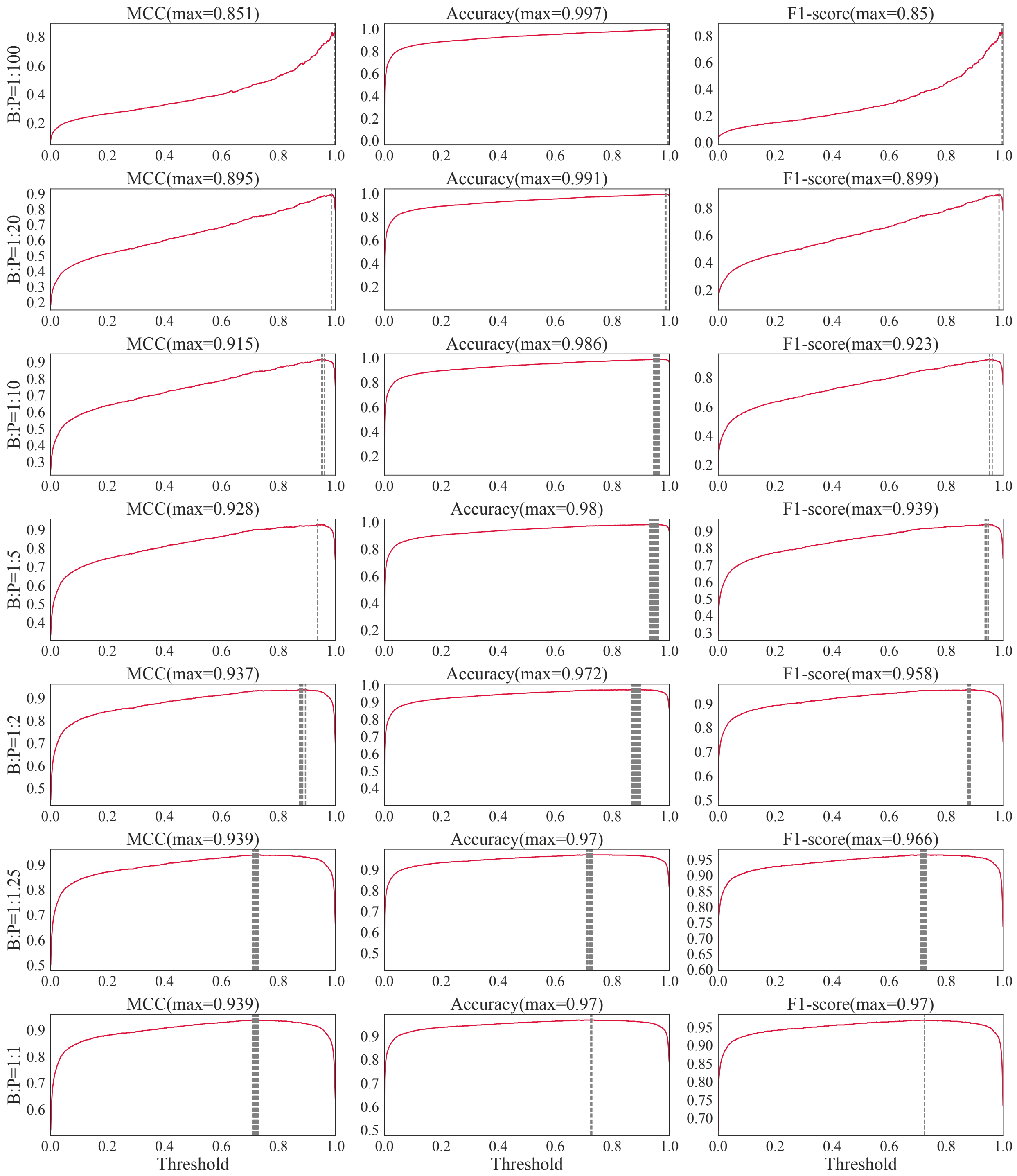

Threshold test of MAGPIE on orthogonal validation set when the number of pathogenic variants is less than benign. We set different ratios of pathogenic variants to make balanced and imbalanced datasets and plot the performance metrics over changes. B and P stand for benign and pathogenic, respectively.

Fig. S9

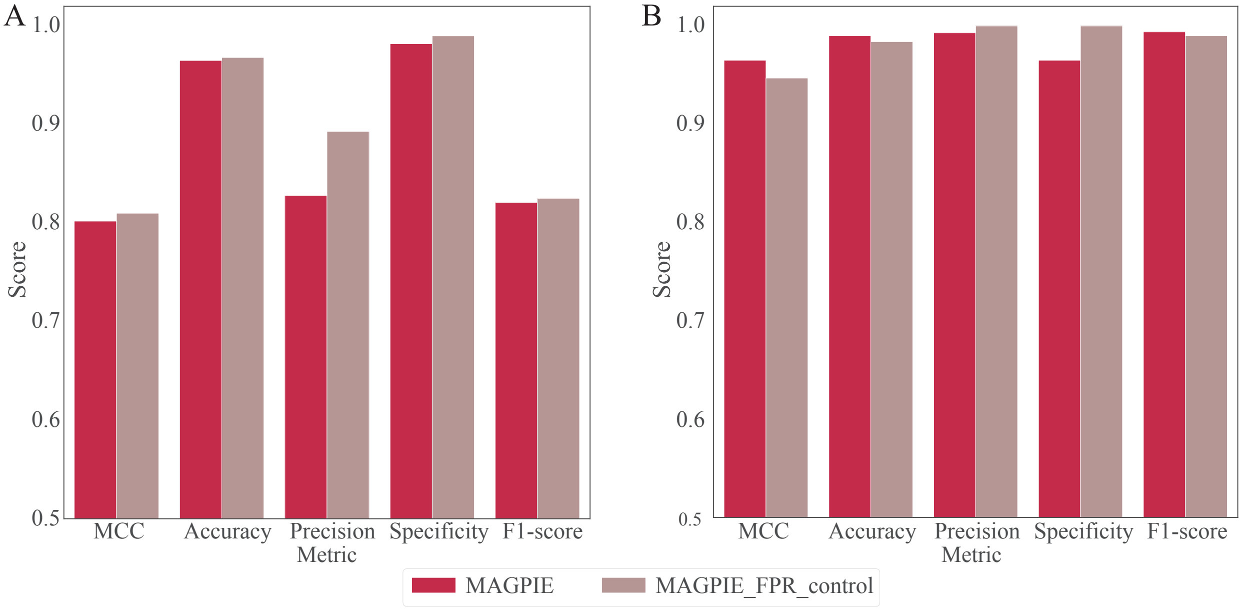

Performance comparison before and after threshold adjustment according to FPR control. We use FPR control to set the threshold for imbalanced datasets, and found that MAGPIE_FPR_control can slightly improve the performance, but using the default threshold of MAGPIE can also achieve great performance in these metrics. **A** Performance comparison on orthogonal validation set. **B** Performance comparison on ACMG guided dataset.

Fig. S10

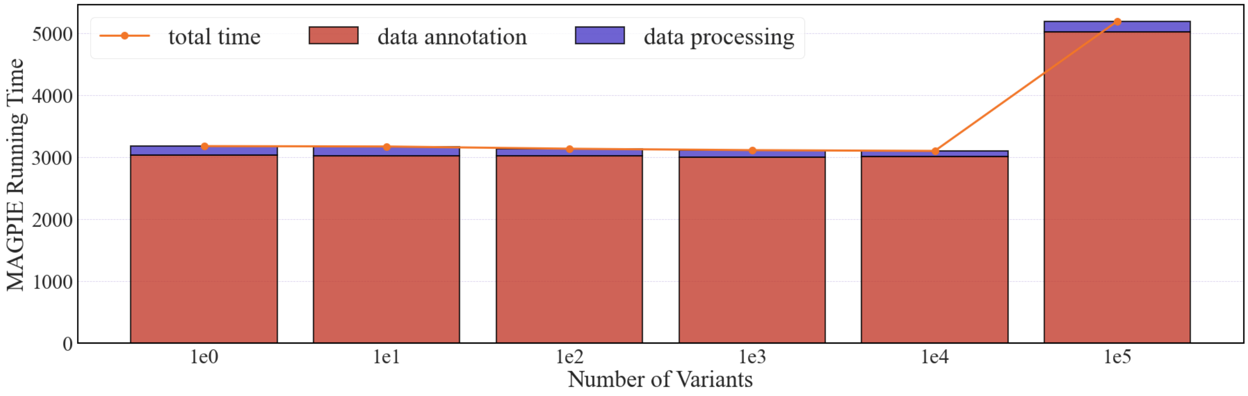

MAGPIE running time. We set different numbers of variants and barplot shows that the MAGPIE running time is stable when number is less than 5e4 because the bottleneck is data annotation. When number of variants is greater than 5e4, running time increases linearly.

Fig. S11

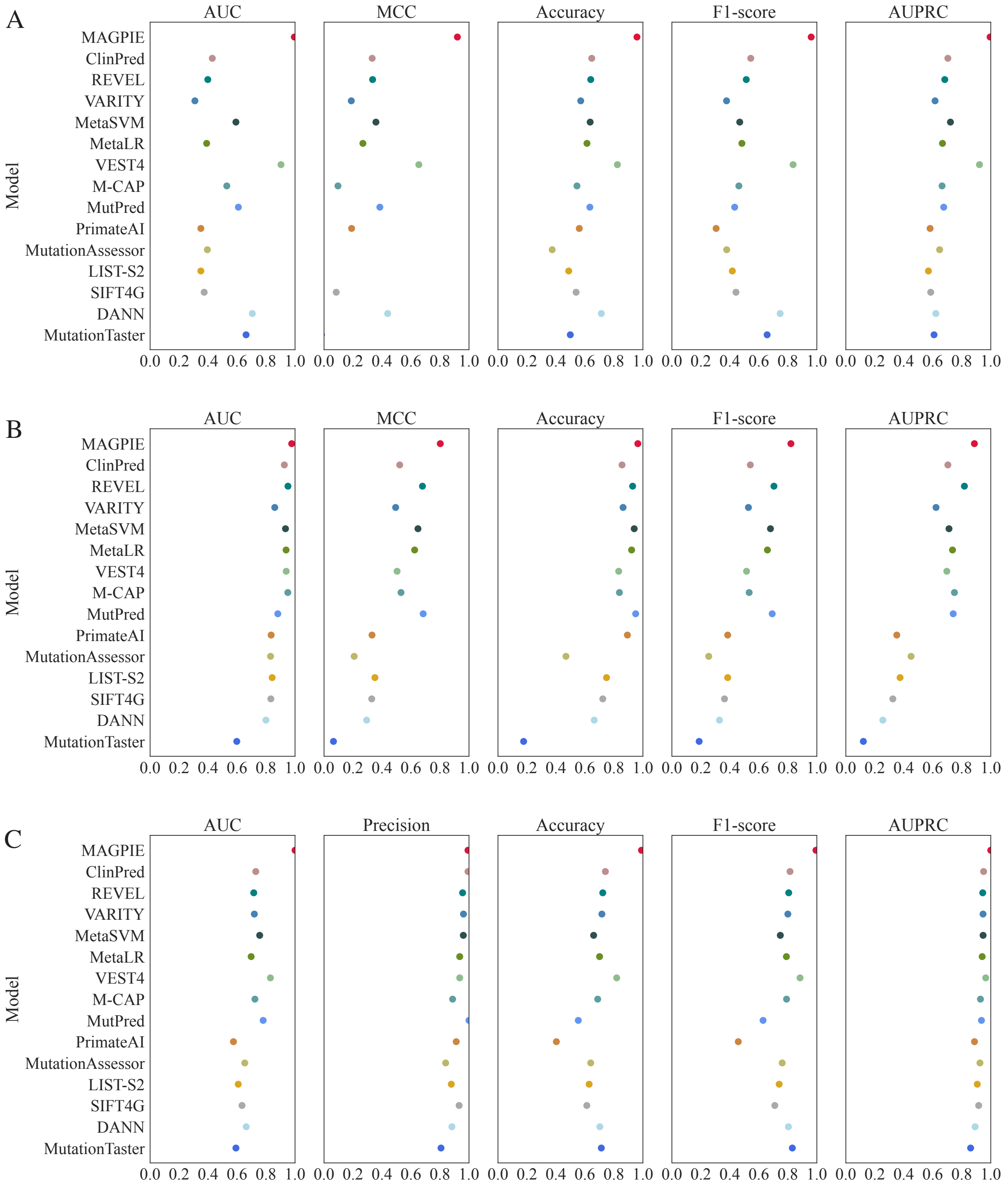

Performance comparison in real-world diagnosis. We compared performances among MAGPIE and other tools considering these unpredictable variants and found all other methods' performance dropped. **A** Performance comparison on independent test set. **B** Performance comparison on orthogonal validation set. **C** Performance comparison on ACMG guided dataset.
